# Supplementary material for: Three dimensional classification of dislocations from single projections
Source: Nat Commun. 2024 Feb 14;15:1356. doi: 10.1038/s41467-024-45642-z (PMC10866901; doi:10.1038/s41467-024-45642-z)
Supplement: Supplementary file 1 — Supplementary Information [file 41467_2024_45642_MOESM1_ESM.pdf]

Three dimensional classification of dislocations from single projections: Supplementary information

Tore Niermann, Laura Niermann, Michael Lehmann

Technische Universität Berlin, Institut für Optik und Atomare Physik, Straße des 17. Juni 135, 10623 Berlin, Germany

(Email: tore.niermann@tu-berlin.de)

SUPPLEMENTARY NOTE 1: APPLICATION TO MECHANICALLY DEFORMED ALUMINUM

The described method was also tested within a dislocation network of a mechanically deformed Aluminum sample as an alternative material system. As typical for face centered materials, perfect dislocations are expected to have Burgers vectors $\mathbf{b}$ corresponding to $\frac{1}{2}\langle 110\rangle$ crystal vectors [1].

The specimen slab was prepared from the bulk with surfaces close to the $(01\bar{1})$ crystal planes by means of focused ion beam milling. The TEM lamella was rotated by roughly 9 degrees from the $[01\bar{1}]$ zone axis into the (200)-systematic row condition. A 4D-STEM dataset was acquired in this orientation using the same instrumentation and parameters as described in the main text with the following exceptions: the dwell time was 50 ms and a roughly 8 times lower beam current was used (for no special reasoning).

An annular dark field (ADF) image calculated from the 4D-STEM dataset is shown in Supplementary Fig. 1a. Within this image several dislocations can be seen as linear features. Furthermore, some fairly coherent precipitates can be noticed as dark and bright round features ranging from 5 to 30 nm diameter. The dislocation marked by the white arrow was selected for subsequent analysis. The direction of the dislocation line is parallel to $[\bar{1}11]$. The resulting (q, x)-plane in Supplementary Fig. 1c was obtained from the 4D-dataset averaging the data points in the red rectangles in Supplementary Fig. 1a and 1b perpendicular to the red arrows. Its x-direction corresponds to the [211]-crystal direction and its q-direction is oriented along the reciprocal space (200)-direction.

Possible Burgers vectors compatible with the $\mathbf{g}\cdot\mathbf{b}$ criterion for this dislocation are $\frac{1}{2}[\pm 1 \pm 1\, 0]$ and $\frac{1}{2}[\pm 1\, 0 \pm 1]$. As described in the Methods section of the main text (q, x)-planes were simulated for these Burgers vectors by means of the propagation of the Darwin-Howie-Whelan equations. The resulting (q, x)-plane for Burgers vectors with positive x-component are shown together with the mean squared errors resulting from comparison of the calculated and experimentally obtained (q, x)-planes in Supplementary Fig. 2. The (q, x)-planes for Burgers vectors with negative x-component can be obtained by simply flipping the q-direction of the presented calculated (q, x)-planes and are not further discussed here, as they obviously differ strongly from the experimental (q, x)-plane.

For an individual Burgers vector the mean squared error maps already show a sufficiently well recognizable global minimum identifying the depth of the dislocation and the specimen thickness, but the type of dislocation cannot be uniquely determined in this case, due to the midplane ambiguity. For the edge-type dislocation with Burgers vectors $\mathbf{b}_2 = \frac{1}{2}[110]$ (Supplementary Fig. 2d) and $\mathbf{b}_4 = \frac{1}{2}[101]$ (Supplementary Fig. 2h) significantly lower mean squared errors (2656 resp. 2728) are observed than for the mixed-type dislocations with Burgers vectors $\mathbf{b}_1 = \frac{1}{2}[1\bar{1}0]$ (Supplementary Fig. 2b; MSE=3024) and $\mathbf{b}_3 = \frac{1}{2}[10\bar{1}]$ (Sup-

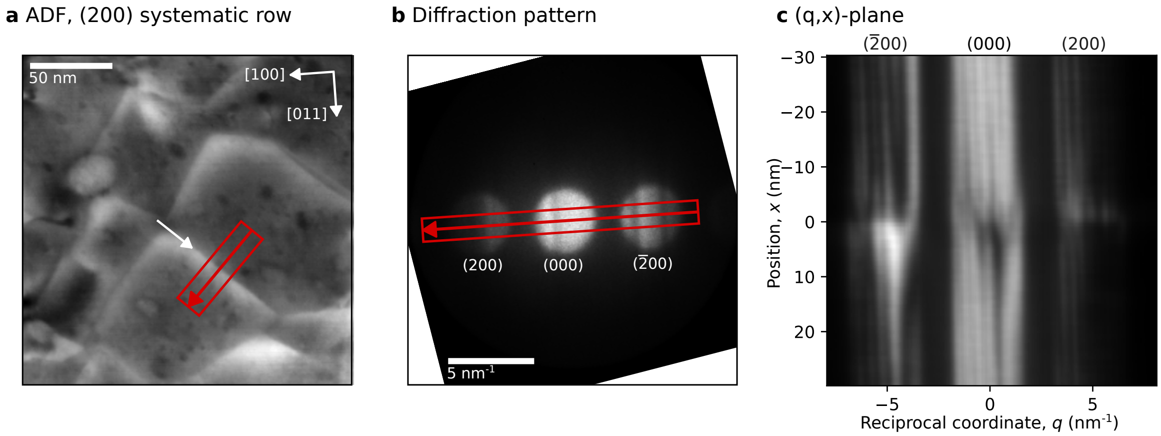

Supplementary Figure 1. **Aluminum sample overview.** **a** Annular dark field (ADF) image in (200) systematic row conditions with evaluated area marked by red rectangle (the red arrow marks the spatial direction x). The white arrow indicates the direction of the dislocation line (parallel to $[\bar{1}11]$) of the evaluated dislocation. The crystal-directions are indicated. **b** Diffraction pattern averaged over all scan coordinates within the red rectangle of **a** (the red arrow marks the reciprocal space direction q). The diffraction pattern display has been flipped and rotated to match the scan coordinate system. **c** Resulting (q, x)-plane.

plementary Fig. 2f; MSE=3150). Thus, the observed dislocation already can be classified as edge-type, while a further discrimination between both edge-type dislocations is not supported by the data. For both edge-type dislocations $\mathbf{b}_2$ and $\mathbf{b}_4$ similar thicknesses of $t = 242\,\mathrm{nm}$ resp. $t = 240\,\mathrm{nm}$ and similar distances of the dislocation from the closest specimen surface have been determined ($\mathbf{b}_2$ is found $d = 20\,\mathrm{nm}$ after the entrance surface while $\mathbf{b}_4$ is found $t - d = 22\,\mathrm{nm}$ before the exit surface).

The Burgers vectors of these two dislocation types just differ by the sign of their component along the electron beam direction (here close to $\mathbf{e}_z \parallel [01\bar{1}]$), i.e. $b_{2,z} \approx -b_{4,z}$. When the small differences in thickness and in distance from the next surfaces between both cases are neglected, the relevant derivative of the displacement field $\mathbf{u}_2(x, z)$ resulting from a $\mathbf{b}_2$ -type dislocation located in a certain distance after the entrance surface and the relevant derivative of the displacement field $\mathbf{u}_4(x, z)$ of a $\mathbf{b}_4$-type dislocation located in a similar distance before the exit surface have a mirror symmetry along the specimen midplane at $z = \frac{t}{2}$ in respect with each other:

$$\frac{\partial}{\partial z} \left(\mathbf{g} \cdot \mathbf{u}_2(x, z)\right) \approx \frac{\partial}{\partial z} \left(\mathbf{g} \cdot \mathbf{u}_4(x, t - z)\right). \tag{1}$$

For a centrosymmetric structure like Aluminum, it can be shown that the diffraction intensities resulting from scattering of these two cases are indeed identical [2]. Thus, no difference in the resulting (q, x)-plots is expected and both cases are indistinguishable in diffraction patterns.

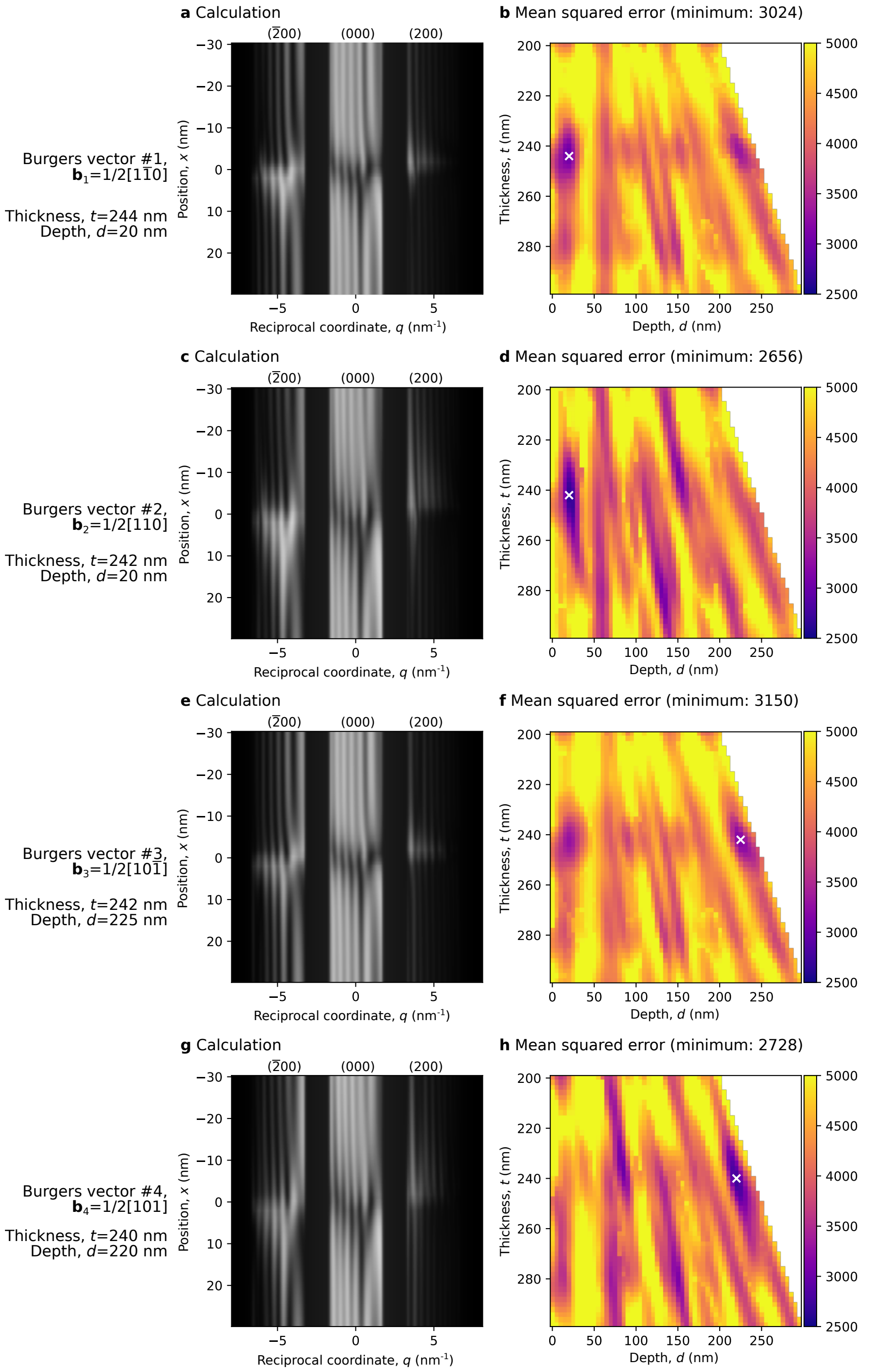

Supplementary Figure 2. **Comparison of calculated (q, x)-planes for different Burgers vectors with the experimentally observed plane (cf. Supplementary Fig. 1c). a, c, e, g** Calculated (q, x)-planes for optimal parameters of thickness t and depth d. **b, d, f, h** Mean squared error maps between experimental and calculated intensities for different values of specimen thickness t and dislocation depth d. **a, b** Data for Burgers vector $\mathbf{b}_1 = \frac{1}{2}[1\bar{1}0]$, $t = 244\,\text{nm}$, $d = 20\,\text{nm}$. **c, d** Data for Burgers vector $\mathbf{b}_2 = \frac{1}{2}[110]$, $t = 242\,\text{nm}$, $d = 20\,\text{nm}$. **e, f** Data for Burgers vector $\mathbf{b}_3 = \frac{1}{2}[10\bar{1}]$, $t = 242\,\text{nm}$, $d = 225\,\text{nm}$. **g, h** Data for Burgers vector $\mathbf{b}_4 = \frac{1}{2}[101]$, $t = 240\,\text{nm}$, $d = 220\,\text{nm}$.

SUPPLEMENTARY NOTE 2: ALTERNATIVE THICKNESS DETERMINATION

Beside the type and position of the dislocation the specimen thickness is a further parameter, which must be determined by the method reported in the main text. In order to verify the obtained thickness, we utilized off-axis electron holography (EH) as an alternative method of thickness determination. With EH it is possible to reconstruct the spatially resolved phase shift $\varphi(x, y)$ the electron wave experienced during its path through the specimen relative to a wave passing through vacuum. When the specimen is oriented away from strongly scattering orientations (kinematic diffraction conditions) this phase shift is given as

$$\varphi(x, y) = \sigma \overline{V} t(x, y), \qquad (2)$$

where σ is an interaction constant (here $\sigma \approx 7.29\ \frac{\mathrm{mrad}}{\mathrm{V\,nm}}$ for 200 kV acceleration voltage), $\overline{V}$ is the mean inner potential of the material, and $t(x, y)$ is the local thickness [3].

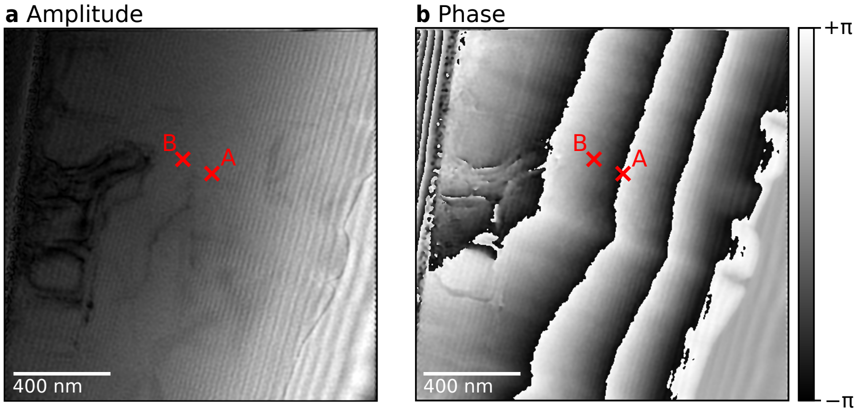

Supplementary Figure 3. **Reconstructed holograms.** **a** Amplitude. **b** Phase. The positions of the dislocations A and B have been marked by the red crosses. Please note the phase wrapping at values of $\pm\pi$. The absolute phase shifts relative to the vacuum area at the positions A and B have been obtained by unwrapping the phase, however the phase wraps have been left in **b** for display purposes. The amplitude and phase images have been rotated to match the specimen orientation in Supplementary Fig. 5.

A series of electron holograms has been acquired using the FEI TITAN 80-300 Berlin Holography Special operated at 200 kV in Lorentz-mode. The specimen was oriented such that bending contours were minimized in a region reaching from the vacuum to the investigated dislocations in order to achieve kinematic diffraction conditions. The series was reconstructed and averaged using the Holoaverage package [4, 5] with a reconstruction aperture of $0.06\mathrm{nm}^{-1}$ radius. The resulting reconstructed amplitudes and phases are shown in Supplementary Fig. 3 (compare Supplementary Fig. 5 for a dark field image). The vacuum region is located at the right hand side, the steep increase in phase on the left hand side corresponds to the sapphire substrate. Furthermore, some Fresnel fringe artifacts are

observable towards the right hand side.

Additionally, CBED patterns where acquired at several positions in thinner specimen areas of 60 nm to 120 nm thickness in (0002) systematic row conditions. The mean inner potential of $\overline{V} = 15.3 \pm 1.0\,\mathrm{V}$ was estimated by relating the observed phase shift at the positions of the CBED patterns with the specimen thickness obtained from comparison of the CBED patterns with simulations. It should be noted, that the value of the mean inner potential is very sensitive to the specimen surfaces [6] and doping [3] (the specimen has no intentional doping in this area). This value of the mean inner potential is within the range of values reported in the literature (e.g. $12 \pm 2\,\mathrm{V}$[7], $13.8 \pm 0.6\,\mathrm{V}$[8], $16.7 \pm 0.3\,\mathrm{V}$ [9]).

The obtained phase shifts at the approx. positions of dislocations A and B are $\varphi_A = 15.55 \pm 0.39\,\mathrm{rad}$ resp. $\varphi_B = 18.49 \pm 0.34\,\mathrm{rad}$, which corresponds to specimen thicknesses of $t_A = 139.5 \pm 9.7\,\mathrm{nm}$ resp. $t_B = 166 \pm 11\,\mathrm{nm}$. These values are within the errors of the thicknesses obtained by the method in the main text, thus support the observed thicknesses.

SUPPLEMENTARY NOTE 3: INFLUENCE OF WINDOW SIZE

For denser dislocation networks, specimens with a higher density of defects, or cases where the specimen properties are not constant over a larger region, it becomes necessary to reduce the spatial extent of the investigated area (represented by the red rectangles in Fig. 1b and 1d of the main text). Here, we investigate how a smaller spatial extent will influence the described method.

We cropped the experimental data of the (q, x)-plane for dislocation A presented in the main text symmetrically around the dislocation. The full spatial extent of the original dataset in x-direction was 89 nm. This extent was reduced to 61, 41, 21, and 11 nm and was compared to simulated (q, x)-planes calculated for these spatial extents. The resulting mean squared error maps for dislocation depth d and specimen thickness t are shown in Supplementary Fig. 4.

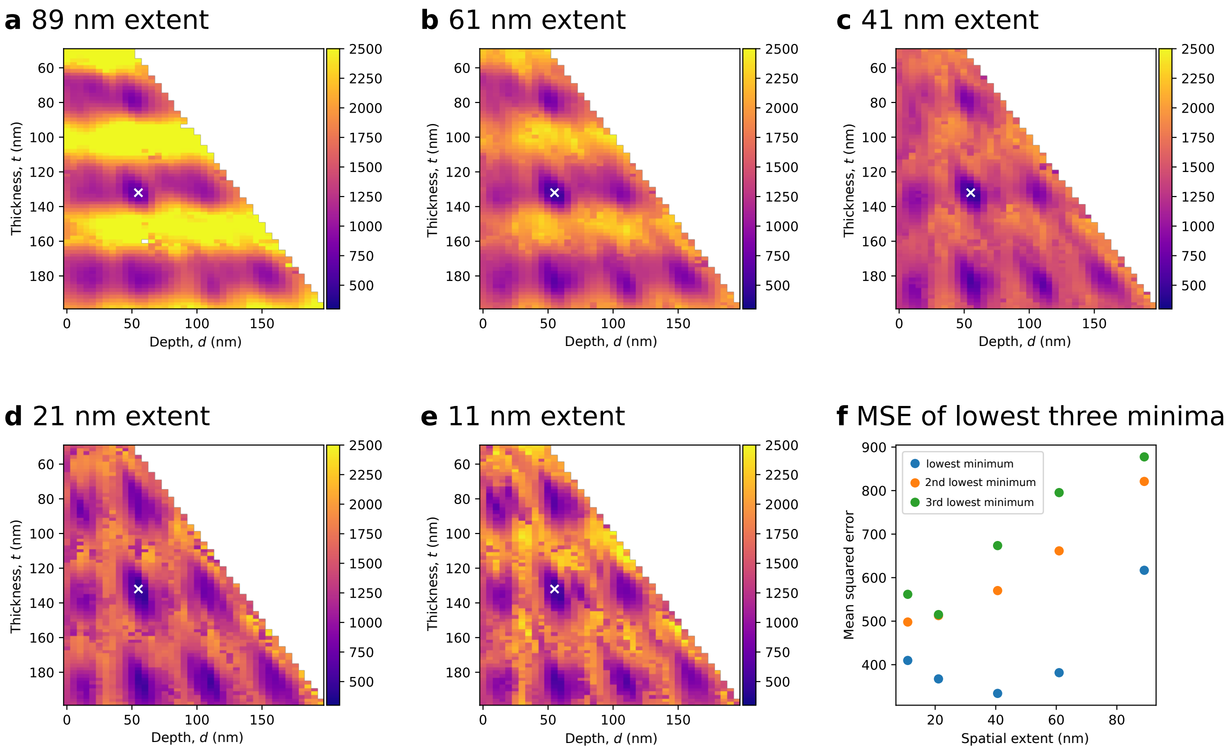

Supplementary Figure 4. **Mean squared errors (MSE) for dislocation A for different spatial extents in x-direction.** **a** 89 nm extent (same extents as in the main text). **b** 61 nm extent. **c** 41 nm extent. **d** 21 nm extent. **e** 11 nm extent. The data of the MSE map in **a** is identical to Fig. 2c of the main text. It is repeated here for convenience with a slightly different color coding to improve comparison with the maps in **b-e** (here between MSE 300 to 2500, compared to MSE 500 to 3000 in Fig. 2c). **f** MSE values for the lowest three minima (blue: global minimum, orange: 2nd lowest minimum, green: 3rd lowest minimum)

For all tested extents the same global minimum at a thickness of $t = 132$ nm and dislo-

cation depth of $d = 55\,\text{nm}$ is identified. Even better MSE values are observed for an spatial extent of 41 nm. Probably, spatial variations of the specimen properties like the observed thickness gradient in this specimen area or the influence of dislocation D as discussed in the main text cause stronger deviations from the calculated data. Also the discrimination between the global minimum and the other minima is stronger in the cases of 41 nm and 61 nm extent. For smaller spatial extents the MSE value at the global minimum as well as the MSE difference to the second lowest minimum worsens, however even for 11 nm extent the second lowest minimum exhibits an MSE value which is still $\approx 25\%$ larger than the value of the optimum.

Overall the described method behaves fairly robust even for spatial region of small extent. However, the required spatial extents for a robust 3D dislocation classification will ultimately depend on the systematic row, the material, and also the thickness, as the features of the (q, x)-plot will become less distinct for thicker specimen, which can be already seen comparing the datasets for dislocation A and B in the main text.

SUPPLEMENTARY NOTE 4: COMPUTATIONAL DEMANDS

The calculation time for the simulation of the $I_g(x, -k; t, d)$ intensity arrays described in the methods section of the main text asymptotically scales with $\mathcal{O}(N_x \times N_k \times (t_{\text{max}}/t_{\text{step}}) \times N_d \times N_g^2)$, where N_x is the number of positions, N_q is the number of lateral wave vectors, and N_d is the number of dislocation depths probed. The number of beams N_g will even enter quadratically, however can be hardly compromised for accuracy reasons. Furthermore t_{max} is the maximal thickness included in the simulation and t_{step} the step size. Separate simulations have to be done for each Burgers vector and dislocation line direction in question, except where the cases are identical due to crystal symmetry. However, often the number of possible (non crystal-symmetric) cases of Burgers vectors is quite small (e.g. 2 for dislocation A, 3 for dislocation B).

We performed the calculations on a desktop workstation equipped with a single AMD Ryzen 9 3900X 12-core processor and 32 GB of RAM. The simulation is CPU limited and runs parallel on the 12 cores. The propagation of the Darwin-Howie-Whelan equations is a very efficient simulation compared to e.g. multislice calculations: the simulation used to find the depth of dislocation A in the main text required 812 seconds with $N_x = 100$, $N_k = 321$, $N_d = 41$, $N_g = 11$, $t_{\text{max}} = 200\,\text{nm}$, and $t_{\text{step}} = 0.1\,\text{nm}$ (for a single Burgers vector). The simulation for a single Burgers vector in the Aluminum case described above required a similar time of 913 seconds with $N_x = 50$, $N_k = 241$, $N_d = 61$, $N_g = 9$, $t_{\text{max}} = 300\,\text{nm}$, and $t_{\text{step}} = 0.1\,\text{nm}$.

SUPPLEMENTARY FIGURES

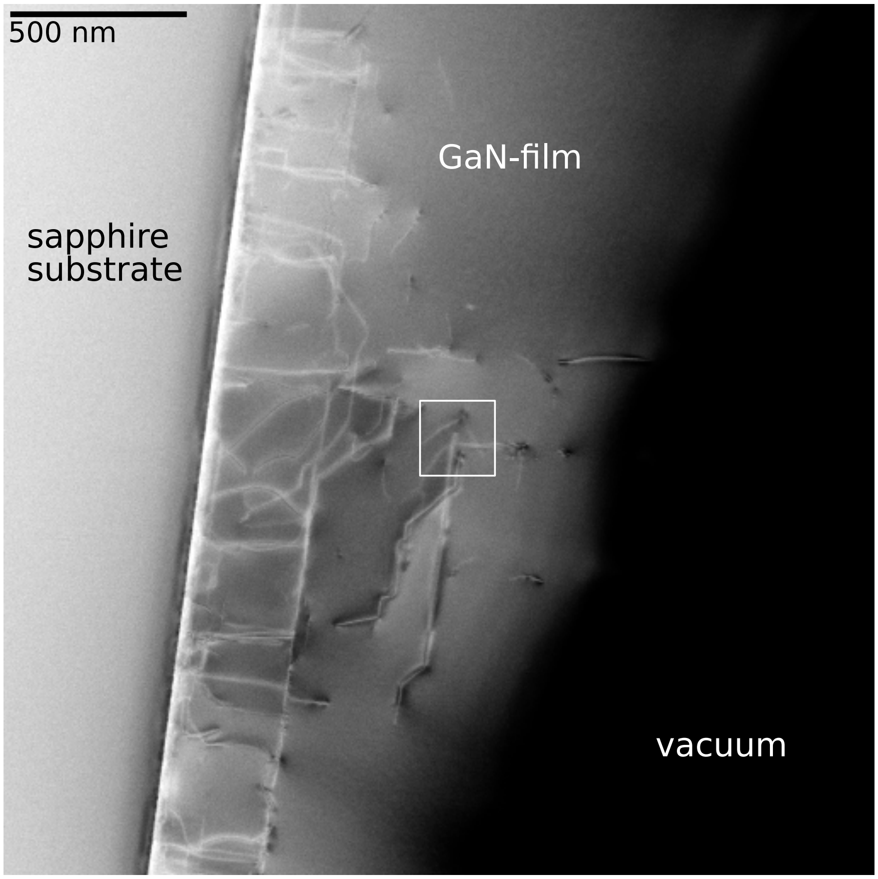

Supplementary Figure 5. **Large area specimen overview.** Annular dark field image with 20 mrad to 103 mrad detection angle, obtained along the $[0\bar{1}10]$ zone axis: Overview of specimen. The sapphire substrate is visible to the left hand side. The region investigated in the main text is marked by the white rectangle.

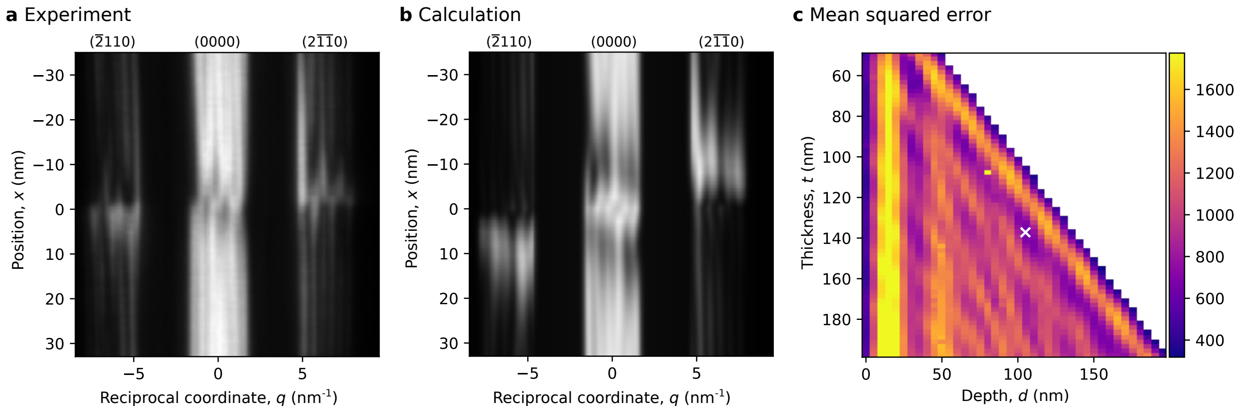

Supplementary Figure 6. **Comparison of (x, q)-planes for dislocation B under $(2\overline{1}\overline{1}0)$-systematic row condition**: **a** Experimental intensities (repeated data from Fig. 3a of the main text for convenience), **b** Simulated intensities for a thickness of $t = 137\,\mathrm{nm}$, a depth of the dislocation core of $d = 105\,\mathrm{nm}$, an incident beam tilt of $\tau = 0.51\,\mathrm{mrad}$, a Burgers vector of $\mathbf{b} = \frac{1}{3}[2\overline{1}\overline{1}0]$, and a line vector parallel to $[14\,\overline{7}\,\overline{7}\,15]$, **c** Mean squared error map between experimental and calculated intensities for different values of specimen thickness t and dislocation depth d; uncolored points indicate untested points or diverged fits, white cross labels parameters used in **b**. In comparison to Fig. 3 of the main text and Supplementary Fig. 7 the achievable mean squared errors are significantly larger for all thicknesses and depths.

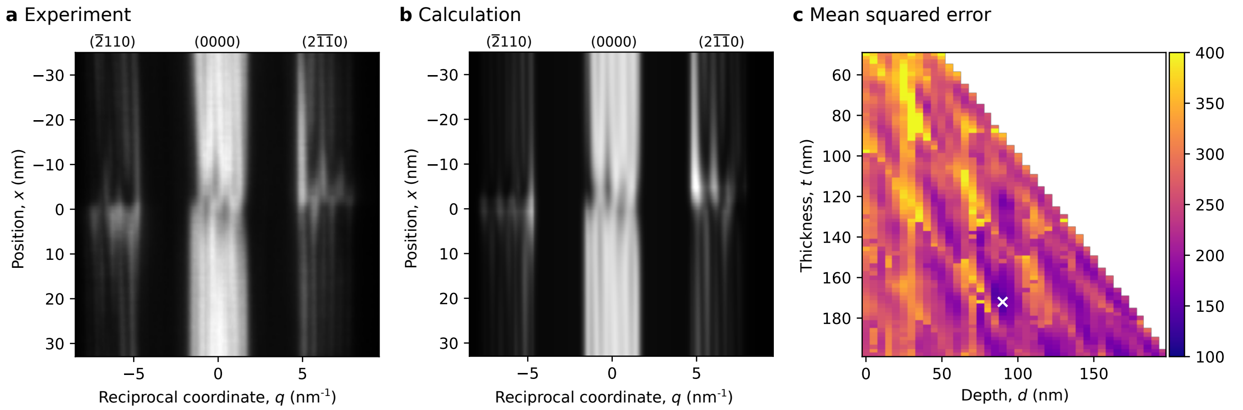

Supplementary Figure 7. **Comparison of (x, q)-planes for dislocation B under $(2\bar{1}\bar{1}0)$-systematic row condition**: **a** Experimental intensities (repeated data from Fig. 3a of the main text for convenience), **b** Simulated intensities for a thickness of $t = 172\,\mathrm{nm}$, a depth of the dislocation core of $d = 90\,\mathrm{nm}$, an incident beam tilt of $\tau = 0.81\,\mathrm{mrad}$, a Burgers vector of $\mathbf{b} = \frac{1}{3}[11\bar{2}0]$, and a line vector parallel to $[14\,\bar{7}\,\bar{7}\,15]$ (optimal parameter set for this Burgers vector), **c** Mean squared error map between experimental and calculated intensities for different values of specimen thickness t and dislocation depth d (uncolored points indicate untested points or diverged fits, white cross labels parameters used in **b**).

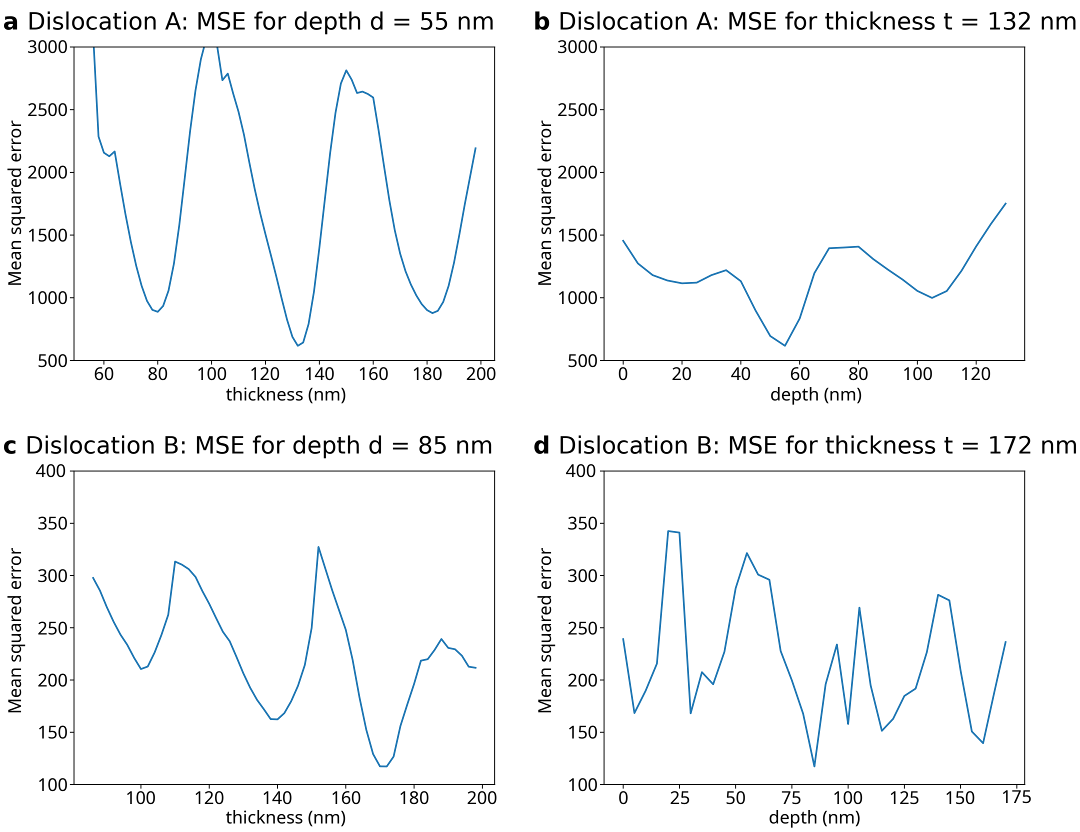

Supplementary Figure 8. **Profiles of the mean squared error (MSE) between experimental and calculated intensities.** **a** Dislocation A: MSE for different values of specimen thickness t and for a constant value of dislocation depth $d = 55$ nm used in the simulation. **b** Dislocation A: MSE for different values of dislocation depth d and for a constant thickness $t = 132\,\text{nm}$ used in the simulation. **c** Dislocation B: MSE for different values of specimen thickness t and for a constant value of dislocation depth $d = 85$ nm used in the simulation. **d** Dislocation B: MSE for different values of dislocation depth d and for a constant thickness $t = 172\,\text{nm}$ used in the simulation. For a full MSE map see Fig. 2c of the main text for dislocation A and Fig. 3c of the main text for dislocation B.

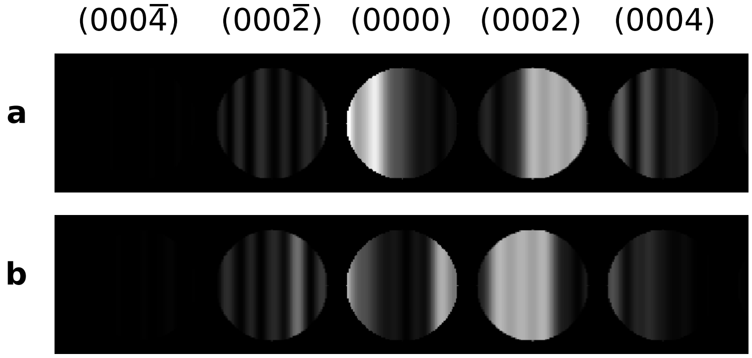

Supplementary Figure 9. **Calculated elastic convergent beam electron diffraction patterns of unstrained GaN. a** Incident beam tilt of $\tau = 5.1$ mrad (cf. Fig. 2a of the main text for $x \approx -40\,\text{nm}$), **b** Incident beam tilt of $\tau = 3.1$ mrad (cf. Fig. 2b of the main text for $x \approx +40\,\text{nm}$). The CBED patterns have been simulated by the Bloch wave method for a specimen thickness of $t = 132$ nm. In contrast to the calculated data shown in the main text, these pattern are not convoluted by the modulation transfer function of the detector, nor an empirical background was added to them.

SUPPLEMENTARY REFERENCES

[1] Hirth, J. P. & Lothe, J. *Theory of Dislocations (2nd Ed.)* (John Wiley & Sons, New York, 1982).

[2] Koprucki, T., Maltsi, A. & Mielke, A. Symmetries in transmission electron microscopy imaging of crystals with strain. *Proceedings of the Royal Society A: Mathematical, Physical and Engineering Sciences* **478**, 20220317 (2022).

[3] Lehmann, M. & Lichte, H. Tutorial on off-axis electron holography. *Microscopy and Microanalysis* **8**, 447–466 (2002).

[4] Niermann, T. *Holoaverage software package, https://github.com/niermann/holoaverage*. URL `https://github.com/niermann/holoaverage`.

[5] Niermann, T. & Lehmann, M. Averaging scheme for atomic resolution off-axis electron holograms. *Micron* **63**, 28–34 (2014).

[6] Pennington, R. S., Boothroyd, C. B. & Dunin-Borkowski, R. E. Surface effects on mean inner potentials studied using density functional theory. *Ultramicroscopy* **159**, 34–45 (2015).

[7] Cherns, D., Mokhtari, H., Jiao, C., Averbeck, R. & Riechert, H. Profiling band structure in GaN devices by electron holography. *Journal of Crystal Growth* **230**, 410–141 (2001).

[8] Tanaka, S., Naito, A., Honda, Y., Sawaki, N. & Ichihashi, M. Application of electron holography to the determination of contact potential difference in an AlGaN/AlN/Si heterostructure. *Journal of Electron Microscopy* **56**, 37–42 (2007).

[9] Wong, A. S. W. *et al.* The mean inner potential of GaN measured from nanowires using off-axis electron holography. *MRS Online Proceedings Library* **892** (2005).
